# Metformin and sulodexide restore cardiac microvascular perfusion capacity in diet-induced obese rats

**DOI:** 10.1186/s12933-017-0525-7

**Published:** 2017-04-11

**Authors:** Judith van Haare, M. Eline Kooi, Jurgen W. G. E. van Teeffelen, Hans Vink, Jos Slenter, Hanneke Cobelens, Gustav J. Strijkers, Dennis Koehn, Mark J. Post, Marc van Bilsen

**Affiliations:** 1 Department of Physiology, Maastricht University, P.O. Box 616, 6200 MD Maastricht, The Netherlands; 2 Department of Radiology and Nuclear Medicine, Maastricht University, P.O. Box 616, 6200 MD Maastricht, The Netherlands; 3 Department of Cardiology, CARIM School for Cardiovascular Diseases, Maastricht University, P.O. Box 616, 6200 MD Maastricht, The Netherlands; 4 Biomedical Engineering and Physics, Academic Medical Center, P.O. Box 22700, 1100 DE Amsterdam, The Netherlands; 5 Pie Medical Imaging, P.O. Box 1132, 6201 BC Maastricht, The Netherlands

**Keywords:** Obesity, Endothelial dysfunction, Myocardial perfusion, Cardiac function, Glycocalyx, Metformin, Sulodexide

## Abstract

**Background:**

Disturbances in coronary microcirculatory function, such as the endothelial glycocalyx, are early hallmarks in the development of obesity and insulin resistance. Accordingly, in the present study myocardial microcirculatory perfusion during rest and stress was assessed following metformin or sulodexide therapy in a rat model of diet-induced obesity. Additionally, the effect of degradation of the glycocalyx on myocardial perfusion was assessed in chow-fed rats.

**Methods:**

Rats were fed a high fat diet (HFD) for 8 weeks and were divided into a group without therapy, and groups that received the anti-diabetic drug metformin or the glycocalyx-stabilizing drug sulodexide in their drinking water during the last 4 weeks of the feeding period. Myocardial microvascular perfusion was determined using first-pass perfusion MRI before and after adenosine infusion. The effect of HFD on microcirculatory properties was also assessed by sidestream darkfield (SDF) imaging of the gastrocnemius muscle. In an acute experimental setting, hyaluronidase was administered to chow-fed control rats to determine the effect of enzymatical degradation of the glycocalyx on myocardial perfusion.

**Results:**

HFD-rats developed central obesity and insulin sensitivity was reduced as evidenced by the marked reduction in insulin-induced phosphorylation of Akt in both cardiac and gastrocnemius muscle. We confirmed our earlier findings that the robust increase in myocardial perfusion in chow-fed rats after an adenosine challenge (+56%, p = 0.002) is blunted in HFD rats (+8%, p = 0.68). In contrast, 4-weeks treatment with metformin or sulodexide partly restored the increase in myocardial perfusion during adenosine infusion in HFD rats (+81%, p = 0.002 and +37%, p = 0.02, respectively). Treating chow-fed rats acutely with hyaluronidase, to enzymatically degrade the glyocalyx, completely blunted the increase in myocardial perfusion during stress.

**Conclusions:**

In early stages of HFD-induced insulin resistance myocardial perfusion becomes compromised, a process that can be countered by treatment with both metformin and sulodexide. The adverse effect of acute glycocalyx degradation and protective effect of long-term sulodexide administration on myocardial perfusion provides indirect evidence, suggesting a role for the glycocalyx in preserving coronary microvascular function in pre-diabetic animals.

## Background

Obesity, in particular abdominal obesity, is associated with insulin resistance and increased cardiovascular risk [1, 2]. Insulin resistance, even in the absence of overt diabetes, promotes the development of coronary macrovascular disease through stimulation of atherogenesis as well as microvascular dysfunction via its impact on various endothelial functions [3, 4]. Microvascular dysfunction in chronically obese subjects is characterized by impaired vasomotor activity, increased vascular permeability, reduced capillary recruitment and rarefaction [5–9]. A recent addition to this array of symptoms is the loss of glycocalyx, a proteoglycan-rich hydrogel that separates the blood from the endothelium and is formed by the endothelium [10–12].

In a previous study, myocardial perfusion reserve, i.e. the relative increase in myocardial perfusion during hyperaemic compared to rest condition, became compromised after feeding rats a high-fat diet (HFD), well before manifestations of diabetes and cardiac structural remodeling were present [13]. Accordingly, cardiac microvascular dysfunction appears to be one of the earliest cardiac maladaptations. Such an endothelial dysfunction may relate to the development of insulin resistance causing an imbalance between vasodilator and vasoconstrictor effects of insulin [14]. This imbalance leads to a reduction in total blood flow to the target tissues [15]. In early stages of insulin resistance, compensatory mechanisms restore blood flow, resulting in an increased basal myocardial perfusion [13]. Thus, microvascular endothelium is a potential therapeutic target.

In the current study, we tested if the drugs metformin and sulodexide were able to restore coronary microvascular perfusion in rats challenged with a HFD [11, 16, 17]. The oral antidiabetic drug metformin (dimethylbiguanide) improves insulin sensitivity primarily via its metabolic actions and is the cornerstone of pharmaco-therapy in type 2 diabetes mellitus [18]. In contrast, sulodexide is a commercially available anti-thrombotic drug, consisting of heparin sulphate (80%) and dermatan sulphate (20%), that has previously been shown to preserve endothelial glycocalyx function [10–12, 19]. To evaluate the involvement of the endothelial glycocalyx in coronary microvascular dysfunction in more detail, rats on a control diet were pretreated with hyaluronidase, an enzyme that rapidly degrades the glycocalyx.

The present findings show that the blunted hyperaemic response in myocardial microvascular perfusion in HFD rats is restored after treatment with both metformin and sulodexide. The observation that sulodexide restored coronary perfusion reserve in HFD rats, whereas hyaluronidase had the opposite effect in control rats, suggests that the glycocalyx may be a therapeutic target to preserve coronary microvascular function in pre-diabetic animals.

## Methods

### Animals and diet

All animal experiments were performed according to a protocol approved by the Animal Ethics Care and Use committee of Maastricht University (AEC protocol number: 2010-122). Ten weeks old male Wistar Hannover rats (body weight 300–324 g; Harlan Laboratories, Horst, The Netherlands) were housed in standard cages with 2 rats per cage, in a temperature-controlled room (~23 °C; 40–60% humidity) under a 12/12 h light/dark cycle at the animal facility of Maastricht University. Five control rats received standard chow (Ssniff GmbH, Soest, Germany, containing on caloric basis 9% fat, 58% carbohydrates, and 33% protein) for 4 weeks. Twenty-four rats received HFD (D12492; Research Diets, New Brunswick, containing on caloric basis 60% fat, 20% carbohydrate, and 20% protein) for 8 weeks. The HFD treated animals were divided into a group without therapy (n = 8), a group that received metformin (0.3 mg/ml; n = 8), and a group that received sulodexide (0.15 mg/ml; n = 8) in their drinking water in the last 4 weeks of the feeding period. All animals received diet and water ad libitum.

Every 2 weeks, body weight was determined and blood pressure was measured by the tail cuff method using a CODA non-invasive blood pressure monitoring system (Kent Scientific, Torrington, CT). Blood sampling of the saphenous vein was performed in the non-fasted, conscious animals to determine glucose and plasma insulin levels. Blood glucose (~5 µl) was measured with a glucose meter (Ascencia Contour, Bayer, Mijdrecht, The Netherlands). Blood was collected from the saphenous vein using 75 µl glass capillary tubes (Hirschmann, Eberstadt, Germany). The capillary tubes were centrifuged, plasma haematocrit levels were determined (MSE, micro-haematocrit reader), and plasma was collected to measure plasma insulin levels with an Ultrasensitive Insulin ELISA kit according to the manufacture protocol (ALPCO Diagnostics, Salem, NH).

In a separate series of experiments of chow-fed rats (n = 9), HFD rats (n = 9), HFD rats that received metformin (n = 9), and HFD rats that received sulodexide (n = 6) during the last 2 weeks of the feeding period (6 weeks), were used to perform an intravenous insulin tolerance test (AEC Protocol Number: 2011-139). The rats were housed under the same conditions as described above.

Finally, in an acute experimental setting either a bolus of 0.5 ml of saline or 0.5 ml of hyaluronidase (1000 U/ml, Type IV-S; Sigma-Aldrich) was intravenously administered in the tail vein of chow-fed control rats (n = 8), 10 min before the start of the MRI measurements, to determine the effect of enzymatically degradation of the glycocalyx on myocardial perfusion [11]. This time point was based on previous studies, where a time-dependent effect of bolus administration of hyaluronidase on impairment of glycocalyx exclusion properties was shown [20, 21].

### MR imaging

At the day of experiment, rats were subcutaneously injected with 0.05 mg/kg of buprenorphine about 30 min before surgery, and were anesthetized with isoflurane (induction chamber: 4.5% isoflurane and 1% oxygen, during experiment: 2% isoflurane and 0.4% oxygen). The animal was put in a prone position and body temperature was maintained at ~37 °C by the use of a heating pad. During anesthesia, a cannula was inserted in the jugular vein for infusion of adenosine for stress measurements during MRI. For the infusion of the contrast agent for MRI, both left and right femoral vein were cannulated. After surgery, the anesthetized rats were positioned prone and horizontally in an animal holder on a heating pad in the magnet. Body temperature was monitored by a rectal probe. ECG electrodes were positioned on the right front leg and left hind leg and connected to an MR compatible small animal monitoring system (SA Instruments, Stony Brook, NY). Respiratory rate was continuously monitored. Magnetic resonance imaging to determine myocardial function and perfusion was performed during baseline conditions and during intravenous adenosine infusion (3 mg/ml; 1.5 ml/h).

#### Cine MRI

Magnetic resonance imaging was performed on a 7.0 Tesla Bruker Biospec 70/30 USR animal scanner (Bruker Biospin, Ettlingen, Germany) equipped with a quadrature volume coil. Sequential multislice short-axis cine MRI was performed to assess systolic function of the left ventricle. First, a bright blood cine MR image with 10 cardiac phases was recorded in 4-chamber view (4CH) using a retrospectively self-gated protocol (IntraGate, Bruker Biospin) as described previously [22]. The following acquisition parameters were used: pulse repetition time (TR) = 8 ms, echo time (TE) = 2.95 ms and flip angle = 10°. The acquired field of view was 50 × 50 mm^2^ with an acquired 256 × 256 matrix size with an imaging time of ~5 min. Perpendicular to the 4CH view, a long axis view (LA) was acquired using the same self-gated protocol. This imaging slice was oriented as a plane through the mitral valve and the apex. Perpendicular to this LA and 4CH view, two short axis slices (SA) were positioned just below the mitral valve and just above the start of the papillary muscles. For both slices, the same self-gated protocol was used.

#### Myocardial first-pass perfusion MR imaging

A time-series of 300 short-axis images was acquired using a first-pass perfusion sequence, so that the first pass of the contrast agent through the heart was captured. A bolus of 150 µl Gadovist (200 mM; Gadobutrol) was injected during the 15th time phase in the femoral vein, while scanning continued. For the mid-ventricular, short-axis first-pass perfusion images, an ECG-triggered, segmented saturation-recovery FISP sequence was used as described in detail elsewhere [23, 24]. A temporal resolution was reached of one image per four heartbeats. The prospective trigger delay δ_SAT_ with respect to the R-peak of the ECG-signal was adjusted to the heart rate and chosen such that the effective saturation time *T*_SAT_ was between 20 and 30 ms. The segment acquisition time *T*_ACQ_ was 25.76 ms. Additionally, the following acquisition parameters were used: TR = 1.61 ms, TE = 0.8 ms, field of view = 6 × 6 cm^2^, acquisition matrix = 64 × 64 (4 segments, acquired over four consecutive heart beats), slice thickness = 3 mm, 0.3 ms Gauss excitation pulse (α = 15°; BW = 9133 Hz) and a 2 ms Gauss saturation pulse (α = 90°; BW = 1370 Hz). Reconstructed pixel size was 469 × 469 µm^2^ after zero-filling to 128 × 128. Centric *k*-space filling was performed to ensure that low spatial frequency information was least influenced by cardiac motion.

### Imaging of skeletal muscle microcirculation

To determine the effect of HFD on glycocalyx barrier properties, the microcirculation of the gastrocnemius was visualised with a Sidestream Dark-Field (SDF) camera after the first-pass perfusion MR imaging. Three measurements were performed in each rat to visualise the microvessels on different positions in the gastrocnemius. The SDF camera is equipped with a 5× magnifying objective lens system-containing probe, imaging the red blood cells (RBCs) in the tissue-embedded microcirculation using green pulsed LED ring illumination [11, 25]. A micromanipulator was used for holding the SDF camera at the same position. Dimensions of the endothelial glycocalyx were estimated by imaging the microcirculation in the gastrocnemius using the SDF camera with integrated software (GlycoCheck BV, Maastricht, The Netherlands) for automatic analysis of the video recordings as previously described [26, 27].

After imaging of the muscle microcirculation the rats were sacrificed. Heart, peri-epididymal and perirenal fat mass were determined by gravimetry.

### Insulin tolerance test

After an overnight fasting (10–12 h), an intravenous insulin tolerance test (IVITT) was performed in a separate set of chow-fed (n = 9), HFD-fed (n = 9), metformin-treated HFD-fed (n = 9) and sulodexide-treated HFD-fed rats (n = 6), to measure insulin-mediated glucose disposal. After analgesia and anesthesia (isoflurane 2%) the femoral vein was cannulated. Baseline blood glucose concentration was measured via tail bleeding and 2 glass capillaries of blood were taken for baseline plasma insulin measurements. Next, rats received a bolus of glucose (1 g/kg, 0.5 g/ml, i.v.) to avoid the development of hypoglycemia after administering the bolus of insulin 30 min later (0.5 U/kg; 0.5 U/ml). Blood glucose and plasma insulin were measured as described above at the indicated time points. Rats were sacrificed at 5 and 30 min after insulin infusion. Heart, gastrocnemius and soleus muscle were isolated, instantly frozen in liquid nitrogen, and stored at −80 °C.

### Western blotting

Tissues were homogenized, sonicated, and centrifuged in ice-cold SET buffer in the presence of phosphatase inhibitor (PhosSTOP, Roche) and protease inhibitor (Complete, Roche). Protein concentrations of the supernatants were determined using a BCA Protein Assay (Micro BCA Protein Assay Kit, Pierce). After electrophoresis samples were transferred to a PVDF membrane (Bio-Rad) and, after washing, incubated with a phospho-Akt (Ser473) rabbit antibody (Cell Signalling Technology, Beverly MA) and subsequently with anti-rabbit secondary antibody (Cell Signalling Technology) and with HRP substrate (SuperSignal West Femto Chemiluminescent Substrate, Pierce). After stripping (Restore Western Blot Stripping Buffer, Pierce) membranes were incubated with an Akt antibody (Cell Signalling Technology). Chemiluminescent signals were detected and quantified using the ChemiDoc XRS system (Bio-Rad). The ratios of pAkt/Akt were used to assess differences in insulin signalling.

### Data analysis

#### Cine MRI

All images were analysed in MRIcro. The end-diastolic volumes (EDV) and end-systolic volume (ESV) of the left ventricle were considered the largest and the smallest areas, respectively, of the LV cavity in each slice. For the analysis of cine MRI, the window width and level were manually adjusted to recognize the internal ventricular morphologic characteristics [22]. For the measurements of LV volumes, the whole cavity was manually selected in MRIcro. The papillary muscle was excluded from the LV volume during analysis. Length (L) of the LV on the LA view was defined as the longest distance from the apex to the valves. To calculate the left ventricular volumes (LVV), the modified Simpson rule (SR) was used as described before [22]. Briefly, ${\text{LVV}} = {\text{A}}_{\text{m}} \times {\text{L}}/ 3 + \left( {{\text{A}}_{\text{m}} + {\text{A}}_{\rho } } \right)/ 2 \times {\text{L}}/ 3 + 1/ 3 \times {\text{A}}_{\rho } \times {\text{L}}/ 3$. (A_m_, cross-sectional area of the LV cavity in the SA plane, ~1–2 mm below the mitral valve; A_ρ_, cross-sectional area of the LV cavity in the SA plane, approximately at the base of the papillary muscles).

Wall thickness of the left ventricle is calculated using the CAAS MRV FARM software package (version 2.1, Pie Medical Imaging, Maastricht, The Netherlands). In the short axis slice just below the mitral valve, the end-diastolic and end-systolic phase was marked. The endocardial and epicardial borders were manually delineated. Wall thickness is determined for the septum and the free wall, calculating the length of a segment between the epicardial and endocardial contours in the systolic and diastolic phase. For each of the segments, the distance between two contours is given in micrometers.

#### MR perfusion analysis

In the first-pass perfusion data sets, manual segmentations were performed within the CAAS MRV FARM software package (version 2.1, Pie Medical Imaging, Maastricht, The Netherlands). Endocardial and epicardial borders were manually delineated. Contours were drawn in one frame of the slice and were copied automatically to the other, in total 300, frames. Contour edits were also copied to all subsequent frames in the slice and a visual check was performed to examine if the contours fit in all the images of the 300 time frames. In case the contours did not fit in an image, the contours were adjusted in that separate image. The LV cavity, or blood pool, was detected automatically if an endocardial contour was available. Papillary muscles were excluded from the LV cavity. To avoid partial volume effects within the myocardial and LV cavity regions of interests (ROI’s), a margin of 1 pixel was kept from the epicardial and endocardial contours.

After segmentation, signal intensity (SI)-time curves were obtained for the LV cavity and the myocardium. The relative upslope was calculated by dividing the maximum upslope of the myocardium by the maximum upslope of the LV cavity curve. The myocardium was divided in 6 segments, for each segment the relative upslope was calculated. For data analysis, the mean of these 6 segments was used. The upslope was calculated within a kernel of 2–3 time points for the LV cavity and 4–5 time points for the myocardium. The number of points within the kernel used for upslope calculations (e.g. 2–3 for the LV cavity and 4–5 for the myocardium) was adjusted for each signal intensity-time curve based on a visual inspection to acquire the best fit of the upslope.

### Imaging of skeletal muscle microcirculation

During video recording, the software automatically identifies all visible microvessels with a diameter between 5 and 25 µm and measurement sites perpendicular to the vessel direction were selected every 10 µm along the length of each microvessel, as described in our previous study [13]. Data acquisition automatically started when image quality was within acceptable range, i.e. good red blood cell (RBC) and background contrast, in focus and without movement of the imaging unit. When data on a minimum number of 3000 measurement sites was obtained, data acquisition automatically stopped. Average duration of data acquisition was ~5 min. Movies consisted of 40 consecutive frames. In each frame, at each measurement site a total of 21 parallel (every ±0.5 µm) intensity profiles were plotted. The RBC column width was automatically determined at each line for all 40 consecutive frames in a movie, revealing a total of 840 RBC column width measurements at a single measurement (21 profiles × 40 frames) [11]. RBC filling percentage was calculated by determining the number of measurement sites that were filled with a red blood cell divided by 840 (total number of measurement sites). Volume was calculated by RBC filling % multiplied by vessel density (number of perfused vessels per observed muscle area).

To calculate the perfused boundary region (PBR), which is defined as the distance between the median RBC column width and the estimated outer edge of the RBC column, the distribution of the RBC column width of each vascular segment is used [13]. The cumulative distribution curve of the RBC column widths was constructed and used to determine median RBC column diameter (D_P50_). The maximum RBC column width is extrapolated from the 25th and 75th percentile values of the RBC column width distribution curve. The PBR is considered to reflect glycocalyx thickness based on the assumption that loss of integrity of the glycocalyx allows for deeper penetration of erythrocytes into the vessel wall, resulting in increased PBR values [28].

### Plasma lipid analysis

Cholesterol and triglyceride levels were measured in plasma samples of HFD rats, metformin- and sulodexide-treated rats. The enzymatic method to determine cholesterol level is based on the cleavage of cholesterol esters by cholesterol esterase to yield free cholesterol and fatty acids. Cholesterol oxidase then catalyzed the oxidation of cholesterol to cholest-4-en-3-one and hydrogen peroxidase. In the presence of peroxidase, the hydrogen peroxide formed affected the oxidative coupling of phenol and 4-aminophenazone to form a red quinone-imine dye. The color intensity of the dye formed is directly proportional to the cholesterol concentration and was determined by measuring the increase in absorbance (Cobas CHO2I and CHO2A, Roche Diagnostics, Mannheim, Germany).

The enzymatic triglycerides assay is based on using a lipoprotein lipase for the rapid and complete hydrolysis of triglycerides to glycerol followed by oxidation to dihydroxyacetone phosphate and hydrogen peroxide. The hydrogen peroxide produced then reacted with 4-aminophenazone and 4-chlorophenol under the catalytic action of peroxidase and a red dye was formed. The color intensity of the red dye formed is directly proportional to the triglyceride concentration and was measured photometrically (Cobas TRIGL, Roche Diagnostics, Mannheim, Germany).

### Statistical analysis

All data were presented as mean ± standard error of the mean (SEM). Statistical differences between the control group, HFD group, metformin- and sulodexide-treated groups during baseline measurements and adenosine infusion, were tested using a repeated measurements analysis of variance (ANOVA) and/or a one-way ANOVA. A post hoc Bonferroni’s multiple comparison test was performed in case a statistically significant difference was found in the ANOVA tests. An unpaired or paired sample *t* test was used to test statistical differences between 2 (paired) groups. A p value of <0.05 was considered statistically significant.

## Results

### Animal characteristics

**Fig. 1:**
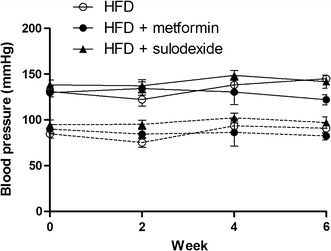
Systolic and diastolic blood pressure of the HFD group and intervention groups. Systolic (*solid lines*) and diastolic (*dashed lines*) blood pressure of the HFD group (n = 8; *open circles*) and intervention groups (n = 8; *filled circles* and n = 8; *triangle*). Statistically significant differences between groups were not observed

Feeding rats a HFD for 8 weeks resulted in marked increases in body weight (+35%, p < 0.0001), and in perirenal (p < 0.0001) and epididymal fat (p = 0.001) depots compared to chow-fed rats (Table 1). Heart weight also increased (p = 0.01), but remained constant when corrected for body weight. The HFD-induced gain in body weight, perirenal and epididymal fat depots, and heart weight was not attenuated in rats that received metformin or sulodexide treatment for the last 4 weeks (Table 1). Blood pressure was not affected in each intervention group (Fig. 1).

**Table 1:** Morphometric data and plasma values of rats in absence or presence of metformin or sulodexide

	Control N = 5	HFD N = 8	Metformin N = 8	Sulodexide N = 7
Body composition				
Body weight (g)	389.6 ± 12.9	528.3 ± 11.6*	532.5 ± 16.2*	555.1 ± 188*
Heart weight (g)	1.00 ± 0.09	1.29 ± 0.05*	1.39 ± 0.08*	1.33 ± 0.04*
Heart weight/body weight (×1000)	2.55 ± 0.15	2.44 ± 0.09	2.61 ± 0.13	2.40 ± 0.06
Perirenal fat (g)	3.44 ± 0.40	14.15 ± 1.57*	15.90 ± 1.52*	16.21 ± 1.27*
Epididymal fat (g)	3.88 ± 0.41	10.19 ± 1.23*	10.85 ± 0.84*	13.00 ± 1.62*
Blood/plasma characteristics				
Haematocrit (%)	50.5 ± 0.5	50.5 ± 1.5	51.3 ± 0.5	48.3 ± 0.4
Total cholesterol (mmol/l)	2.14 ± 0.22	2.86 ± 0.14*	2.58 ± 0.21	2.79 ± 0.05*
Triglycerides (mmol/l)	0.76 ± 0.09	0.97 ± 0.18	0.71 ± 0.11	0.96 ± 0.21
Fasted blood glucose (mmol/l)^a^	6.66 ± 0.37	7.48 ± 0.57	7.42 ± 0.50	7.00 ± 0.60
Fasted insulin (ng/ml)^a^	0.16 ± 0.01	0.76 ± 0.13*	0.59 ± 0.10*	0.30 ± 0.06^#^

Data are expressed as mean ± SEM

*HFD* high fat diet

* p < 0.05 vs. control group, ^#^ p < 0.05 vs. HFD group

^a^Separate series of experiments (control n = 9, HFD n = 9, metformin n = 9, sulodexide n = 6)

HFD, in the absence or presence of metformin or sulodexide, did not affect haematocrit, blood triglyceride levels and fasting glucose levels compared to chow-fed control rats (Table 1). Total cholesterol level was increased in HFD rats and sulodexide-treated rats (p = 0.04). Fasting insulin levels were markedly increased in HFD rats compared to control rats (p = 0.001). Sulodexide treatment, however, significantly decreased insulin levels compared to HFD rats (p = 0.04).

**Fig. 2:**
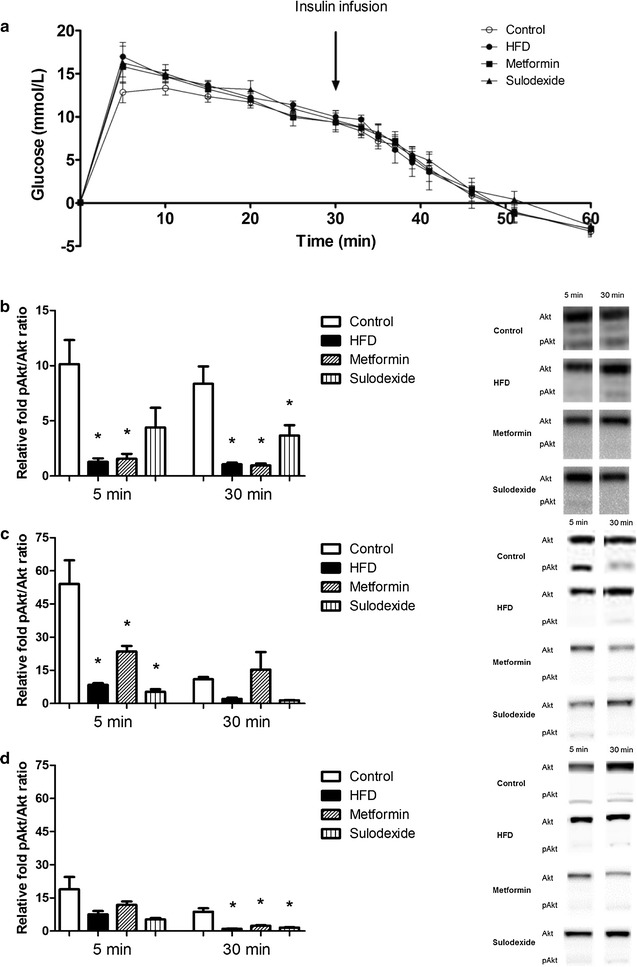
Intravenous insulin tolerance test (IVITT) in control, HFD, and metformin- and sulodexide-treated rats. **a** Glucose level was corrected for baseline glucose level and time indicates the time after glucose infusion. No differences in glucose level were observed between control rats (n = 9) HFD rats (n = 9), HFD rats that received metformin (n = 9) and HFD rats treated with sulodexide (n = 6). Ventricular tissues (**b**), gastrocnemius muscle (**c**) and soleus muscle (**d**) were isolated at t = 5 and t = 30 min (n = 3 per time point) after the insulin injection. For all isolated tissues, representative Western Blots are shown. Data are expressed as mean ± SEM, *p < 0.05 vs. control group. ^#^p < 0.05 vs. HFD group

The insulin tolerance test revealed no significant differences between control rats, HFD rats, and metformin- and sulodexide-treated rats (Fig. 2a), suggesting the absence of overt diabetes or insulin resistance. However, at the tissue level the insulin-dependent increase in Akt phosphorylation, reflecting tissue insulin sensitivity, was markedly reduced in the heart, soleus, and gastrocnemius muscle of HFD rats, compared to control rats (Fig. 2b–d). Metformin did not restore insulin-induced Akt phosphorylation in cardiac muscle or gastrocnemius muscle, compared to HFD rats. In spite of the reduced insulin levels, sulodexide treatment did not improve the insulin-induced Akt-phosphorylation in cardiac muscle or gastrocnemius muscle.

### Cardiac function

Cardiac function under baseline conditions, as measured by cine MRI, was comparable for all four groups (Table 2). Heart rate dropped significantly in all four groups as a consequence of adenosine infusion. During adenosine infusion, end-diastolic left ventricular volume (LV) was significantly increased in the HFD rats and the sulodexide-treated rats (p = 0.0008 and p = 0.04, respectively). This only led to a significant increase in stroke volume (SV) in the HFD rats (p = 0.003). The metformin-treated rats showed a significant decrease in end-systolic LV volume compared to the HFD rats (p = 0.03), resulting in an increased ejection fraction as a consequence of adenosine infusion (p = 0.03). In conclusion, metformin seems to improve cardiac function during adenosine infusion by significantly increasing the ejection fraction.

**Table 2:** Left ventricular function determined by cine MRI after 8 weeks of HFD and metformin or sulodexide treatment

	Baseline				Adenosine			
	Control N = 5	HFD N = 8	Metformin N = 8	Sulodexide N = 7	Control N = 5	HFD N = 8	Metformin N = 8	Sulodexide N = 7
Heart rate (bpm)	384 ± 17	397 ± 10	428 ± 27	386 ± 7	327 ± 21^†^	333 ± 23^†^	361 ± 30^†^	317 ± 14^†^
EDV (µl)	316 ± 33	285 ± 29	294 ± 23	324 ± 48	348 ± 30	394 ± 25^†^	328 ± 39	400 ± 47^†^
ESV (µl)	83 ± 18	71 ± 7	72 ± 16	46 ± 11	55 ± 8	87 ± 14	45 ± 11^#^	61 ± 14^†^
SV (µl)	233 ± 42	214 ± 27	222 ± 32	278 ± 53	294 ± 27	306 ± 32^†^	282 ± 45	339 ± 55
EF (%)	72 ± 5	74 ± 2	74 ± 6	82 ± 6	84 ± 2	77 ± 4	84 ± 5^†^	83 ± 6

Data are expressed as mean ± SEM

*HFD* high fat diet, *EDV* end-diastolic volume, *SV* stroke volume, *EF* ejection fraction

* p < 0.05 vs. control group, ^#^ p < 0.05 vs. HFD group, ^†^ p < 0.05 adenosine effect. No significant differences were found for the intervention groups HFD, metformin and sulodexide compared to the control group

### Myocardial microvascular perfusion

**Fig. 3:**
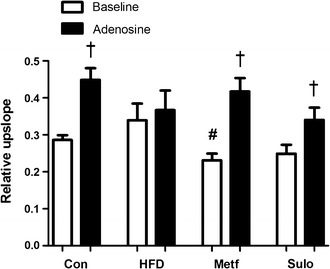
Myocardial perfusion measurements with first-pass perfusion MR imaging. Semi-quantitative myocardial perfusion values (relative upslope) in LV myocardium of control rats (n = 5), HFD rats (n = 8), metformin-treated rats (n = 8) and sulodexide treated rats (n = 7) are presented duringbaseline conditions and adenosine infusion. Data are expressed as mean ± SEM. *p < 0.05 vs. control group, ^#^p < 0.05 vs. HFD group, ^†^p < 0.05 adenosine effect

The effect of metformin and sulodexide treatment in HFD rats on coronary microvascular perfusion was assessed by contrast-enhanced first-pass perfusion MRI. In control rats, adenosine infusion resulted in a robust increase in the relative upslope of the signal intensity, a semi-quantitative MRI parameter for myocardial perfusion (+56%, p = 0.002; Fig. 3). In rats on a HFD for 8 weeks, the adenosine response was completely blunted, (+8%, p = 0.68). In contrast, in the metformin-treated HFD rats the adenosine-induced increase in myocardial perfusion (+81%; p = 0.002) was restored to the level observed in chow-fed control rats. Interestingly, the sulodexide-treated rats also showed a significant increase (+37%; p = 0.02) in myocardial microvascular perfusion during adenosine infusion.

### Glycocalyx properties

**Fig. 4:**
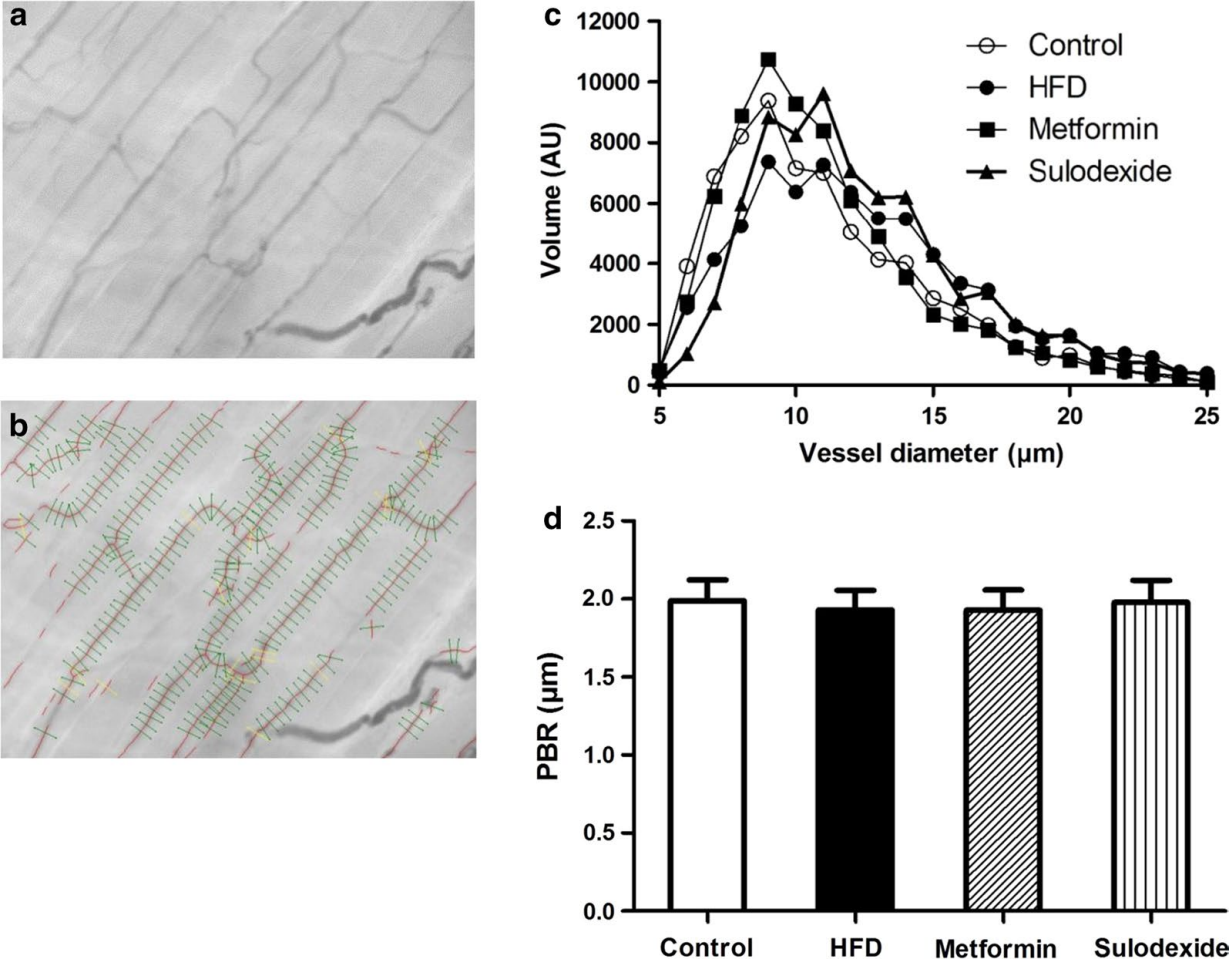
Imaging of the gastrocnemius muscle microcirculation to measure glycocalyx barrier properties. **a** Example of a single SDF image of rat gastrocnemius microcirculation. **b** In each microvessel that fitted the criteria of analysis (**a**) in focus, **b** had a sufficient length and were not close to bifurcation, and **c** were continuously filled with RBCs and did not contain plasma gaps) lines were placed ± every 10 µm perpendicular to the vessel direction (as shown by lines in image). **c** Microvascular volume was determined for all vessel diameters (5–25 µm) for control rats (n = 5), HFD rats (n = 8), metformin-treated rats (n = 8) and sulodexide-treated rats (n = 8) and are not significantly different (p = 0.918 and p = 0.812, respectively). **d** No significant differences are found between the PBR of control rats (1.99 ± 0.13 µm), HFD rats (1.93 ± 0.12 µm), metformin-treated rats (1.93 ± 0.13 µm) and sulodexide-treated rats (1.98 ± 0.14 µm). Data are expressed as mean ± SEM, *p < 0.05 vs. control group, ^#^p < 0.05 vs. HFD group

To look more specifically at the glycocalyx, we performed sidestream darkfield (SDF) imaging of the skeletal muscle microcirculation (Fig. 4a, b). In the gastrocnemius muscle the percentage of perfused microvessels (72% for control rats, 72% for HFD rats, 70 and 81% for metformin and sulodexide-treated rats) and number of perfused microvessels (820 ± 57, 891 ± 89, 900 ± 78, and 947 ± 62 microvessels/mm^2^; p = 0.73) as assessed by SDF imaging, did not differ between the 4 groups. The PBR of HFD rats, metformin- and sulodexide-treated rats amounted to 1.93 ± 0.12, 1.93 ± 0.13 and 1.98 ± 0.14 µm, respectively, and was comparable to the PBR of chow-fed rats (1.99 ± 0.13 µm, p = 0.98) (Fig. 4d). Both metformin and sulodexide treatment did not affect the glycocalyx barrier properties as measured by SDF in peripheral tissues.

### Acute hyaluronidase experiments

In an acute experimental setting, a bolus of hyaluronidase was administered to control rats to provoke loss of glycocalyx [11]. Left ventricular function was not affected as a consequence of hyaluronidase administration (Table 3). During adenosine infusion, heart rate significantly decreased in hyaluronidase-treated rats (p = 0.0004).

**Table 3:** Left ventricular function determined by cine MRI in control rats and hyaluronidase-treated rats

	Control		Hyaluronidase	
	Baseline N = 5	Adenosine	Baseline N = 6	Adenosine
Heart rate (bpm)	384 ± 17	327 ± 21^†^	425 ± 24	334 ± 19^†^
EDV (µl)	316 ± 33	348 ± 30	232 ± 21	273 ± 38
ESV (µl)	83 ± 18	55 ± 8	64 ± 6	74 ± 13
SV (µl)	233 ± 42	294 ± 27	168 ± 17	199 ± 44
EF (%)	72 ± 5	84 ± 2	72 ± 2	71 ± 6

Data are expressed as mean ± SEM

*EDV* end-diastolic volume, *SV* stroke volume, *EF* ejection fraction

* p < 0.05 vs. control group, ^†^ p < 0.05 adenosine effect. No significant differences were found for the intervention group hyaluronidase compared to the control group

**Fig. 5:**
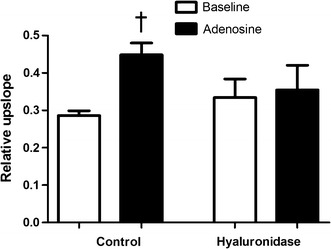
Myocardial perfusion measurements with first-pass perfusion MR imaging. Semi-quantitative myocardial perfusion values (relative upslope) in LV myocardium of control rats (n = 5) and hyaluronidase treated rats (n = 6) are presented during baseline conditions and adenosine infusion. Data are expressed as mean ± SEM. *p < 0.05 vs. control group, ^†^p < 0.05 adenosine effect

Administration of hyaluronidase resulted in a blunted adenosine response of the relative upslope (Fig. 5), thereby suggesting that an intact microvascular glycocalyx is required for regulation of coronary microvascular perfusion.

**Fig. 6:**
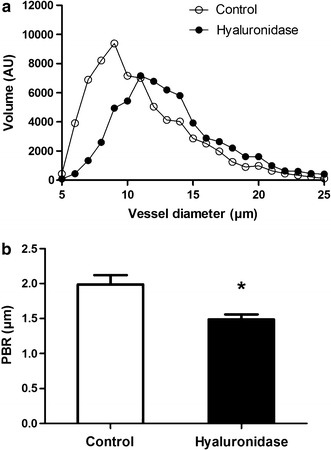
Imaging of the gastrocnemius muscle microcirculation to measure glycocalyx barrier properties. **a** Microvascular volume as a function of vessel diameter was determined for all vessel diameters (5–25 µm) for control rats (n = 5) and hyaluronidase treated rats (n = 6) and are not significantly different (p = 0.579). **b** The perfused boundary region (PBR) of hyaluronidase treated rats was significantly lower (p = 0.04) after hyaluronidase injection than the PBR of control rats (1.49 ± 0.07 vs. 1.99 ± 0.13 µm, respectively). Data are expressed as mean ± SEM, *p < 0.05 vs. control group

The percentage of perfused microvessels in the gastrocnemius muscle (85%) and number of perfused microvessels (705 ± 73 microvessels/mm^2^, p = 0.26) did not significantly differ from control rats. However, the PBR was significantly decreased in hyaluronidase treated rats (−25%, p = 0.04) (Fig. 6b). The collective findings suggest that degradation of the glycocalyx by a bolus of hyaluronidase affected coronary microvascular perfusion capacity, but unexpectedly did not decrease glycocalyx volume in the microvessels of the gastrocnemius muscle.

## Discussion

In this study, we showed that treatment with the insulin-sensitizing drug metformin and the glycocalyx-mimetic sulodexide for 4 weeks improves the coronary microvascular perfusion in rats on a high fat diet. These data suggest that metformin and sulodexide reverse coronary microvascular dysfunction in pre-diabetic conditions.

### Diet-induced obesity and insulin sensitivity

It has been extensively shown that HFD induces abdominal adiposity and variable levels of insulin resistance. The present study confirms previous findings that a HFD challenge leads to an increase in abdominal fat depots, a rise in serum total cholesterol levels, and reduced insulin sensitivity in the cardiac muscle [13]. The changes in adipose tissue and rise in circulating free fatty acids (FFA’s) may result in release of pro-inflammatory cytokines like tumor necrosis factor-alpha (TNF-α), interleukin-6 (IL-6), transforming growth factor-β (TGF-β) and adipokines such as leptin and adiponectin [29–32]. Circulating cytokines, adipokines and non-esterified fatty acids (NEFAs) can affect beta-cell function directly and induce insulin resistance [29–31]. Thus, there is a clear link between obesity, inflammation, and insulin resistance [31, 32].

In the current study, we tested the effects of the anti-diabetic drug metformin and the anti-thrombotic drug sulodexide. Treating HFD rats with either metformin or sulodexide for 4 weeks did not reduce total body mass or adipose tissue mass. Notably, metformin did not lower plasma glucose and insulin levels, although there was a tendency to lower plasma triglyceride levels and to reduced insulin-mediated Akt phosphorylation in the heart and skeletal muscle. The absence of significant effects is most likely related to the relatively low dose of metformin applied in the present study. Indeed, higher metformin doses and longer treatment periods have been shown to increase insulin sensitivity, lower insulin levels, and normalize the lipid profile [18, 33–36]. The chosen dose was based on a previous study from our group, in which the effect of metformin on the glycocalyx properties was examined in severely diabetic db/db mice [34]. The HFD rat model is much milder in that it shows no overt aberration of glucose metabolism. As a consequence the effect of metformin on systemic glucose homeostasis is expected to be limited. Nonetheless, the effect of metformin on cardiac perfusion was clear, indicating that effective plasma levels were reached.

Since we found significantly lower fasting insulin levels in rats treated with sulodexide, this agent may affect the early stage of insulin resistance in high fat diet. Sulodexide is a blend of two glycosaminoglycan (GAG) entities, heparin (HP; 80%) and dermatan sulfate (DS; 20%) [37, 38]. These components regulate the activity of several proteins, including cytokines, growth factors, enzymes, adhesion molecules and modulate leukocytes and macrophages [37, 38]. More specifically, sulodexide inhibits leukocyte activation and the release of TNF-α, TGF-β, IL-6 and several matrix metalloproteinases, which are involved in the destruction of the extracellular matrix [37, 39]. All these anti-inflammatory actions may diminish inflammation-mediated insulin resistance. Our results are in line with a study of Eskens et al. who showed that short-term treatment with sulodexide improves insulin sensitivity in diet-induced obese mice [40]. Sulodexide improved blood glucose levels during an intraperitoneal glucose tolerance test (IPGTT) in mice that were fed a HFD for 6 weeks and received sulodexide in the last 2 weeks of the feeding period. In mice that were fed a HFD for 18 weeks, the insulin resistance index was improved after 2 weeks of sulodexide treatment [40]. Sulodexide could thus be a promising therapeutic agent for patients developing insulin resistance as a consequence of obesity.

### Myocardial microvascular perfusion

HFD blunted the hyperaemic response to adenosine in myocardial perfusion. These results confirm the findings in our previous study, where we saw a robust 40% rise in coronary perfusion in chow-fed rats as a result of adenosine infusion, reflecting an adequate hyperemic response. In contrast, in the myocardium of rats fed a HFD for 6 weeks the hyperemic response was completely blunted [13].

Both metformin and sulodexide restored the comprised myocardial microvascular perfusion in HFD rats, comparable to the level observed in chow-fed control rats. Especially metformin treatment led to a robust adenosine-induced increase in myocardial perfusion (+81%). Metformin provides cardiovascular protection related to favorable actions on endothelial function, lipid metabolism, vascular smooth-muscle and cardiomyocyte intracellular calcium handling, hypercoagulation, and platelet hyperactivity [36]. Metformin has also been shown to have direct vascular effects by increasing NO bioavailability, thereby enhancing NO-dependent relaxation [35, 41, 42]. It was also demonstrated that impaired acetylcholine-induced relaxation in insulin resistant rats, was reversed to control levels after 2 weeks of metformin treatment [35]. Taken together, it is likely that metformin improved cardiac perfusion by restoring balance between vasoconstrictor and vasodilator effects of modest insulin resistance. Sulodexide treatment led to a +37% adenosine-induced increase in myocardial perfusion. Sulodexide is a glycocalyx stabilizer and may therefore improve functions of the glycocalyx in diet-induced obese rats [19]. Enhanced functions of the glycocalyx may alleviate endothelial dysfunction, and thereby improve myocardial perfusion. In accordance with our results, Eskens et al. [40] showed improved insulin sensitivity in diet-induced obese mice after short-term treatment with sulodexide.

To further investigate the role of the glycocalyx in coronary microvascular dysfunction, the effect of acute degradation of the glycocalyx on myocardial perfusion was assessed in chow-fed rats.

A blunted adenosine response was observed in chow-fed rats that received a bolus of hyaluronidase, indicating that an intact glycocalyx is required for normal regulation of coronary microvascular perfusion. Since glycocalyx loss is an early event in the development of endothelial dysfunction and insulin resistance [43], the glycocalyx is a potential therapeutic target. The anti-thrombotic drug sulodexide improved myocardial microvascular perfusion. This drug preserves glycocalyx function and that effect could contribute to improved perfusion [10–12, 19]. However, we were not able to directly establish an effect of sulodexide on glycocalyx volume in our rat model as assessed by sidestream darkfield (SDF) imaging of skeletal muscle microcirculation. It is possible that the effect of sulodexide on the glycocalyx is more subtle in that its biochemical composition is changed without changing its volume. The glycocalyx is a highly complex set of hyaluronic acid and heparansulfates with a wide variety of functions, the anti-thrombin activity being just one of them. Accordingly, it cannot be excluded that the anti-thrombin activity itself or the reported anti-inflammatory effect [37, 38] results in coronary vasodilation and improve cardiac perfusion.

Changes in myocardial microvascular perfusion did not lead to changes in cardiac function. This was expected, since the absence of the hyperaemic response in the myocardium of HFD rats appeared to be due largely to an already increased coronary perfusion at baseline.

An alternative explanation for the improved myocardial hyperemic perfusion response by metformin and sulodexide is based on metabolic shift that the myocardium undergoes from a glucose to a fatty acid substrate as a result of a decreased cardiac insulin sensitivity [13]. Cardiac efficiency will decrease under these conditions and the myocardium in diet-induced obese rats may compensate for this diminished cardiac efficiency by increasing basal myocardial perfusion. Both metformin and sulodexide are able to increase cardiac efficiency by improving insulin sensitivity, which may explain a restored basal myocardial perfusion.

In the present study, we determined the Perfused Boundary Region (PBR) of small vessels by SDF imaging to probe glycocalyx properties of the gastrocnemius muscle. The concept of PBR is based on the notion that loss of glycocalyx allows for deeper penetration of erythrocytes into the endothelial lining [28]. Hence, the PBR provides an indirect measure of the glycocalyx and is not identical to the anatomical thickness of the glycocalyx. In a patient study Lee et al. observed a strong association between changes in PBR and estimates of microvascular perfusion in the sublingual microcirculation in lean, overweight and obese participants [27]. In contrast, Amraoui et al. reported no differences in PBR between healthy volunteers and patients at low or high risk of CVD [26]. One of the limitations of the SDF imaging-based PBR measurements mentioned by the authors was that the method has not yet been validated against an alternative technique for glycocalyx dimension measurement.

Unlike a previous study [11] and despite the observed changes in coronary microvascular perfusion, we observed a decrease, instead of an increase, in the PBR in the microvessels of the gastrocnemius muscle after enzymatic degradation of the glycocalyx with hyaluronidase. It is possible that this is related to the experimental protocol where, because of the intervening MRI, SDF measurements were performed about 2.5 h after hyaluronidase treatment, while earlier studies showed optimal effects of hyaluronidase treatments within 1 h [20, 21].

In our rats we observed no changes in microvascular volume and PBR in the gastrocnemius response to HFD and drug treatment [13]. Notably, Eskens et al. found disturbances in glycocalyx barrier properties in db/db mice that were partially alleviated by a 2 week metformin treatment [34]. In that study glycocalyx barrier properties were assessed at the whole body level by determining the initial vascular distribution volume and clearance of 70 kDa versus 40 kDa dextrans. The difference in animal model (severity of diabetes) and methods to probe the glycocalyx may well explain the differences in outcome between the present study and the study of Eskens et al. [34].

Collectively, the present results indicate that SDF measurements in the gastrocnemius muscle do not follow cardiac perfusion measurements, which is a limitation of the method. Currently, non-invasive techniques, like SDF imaging, to probe the coronary microvascular glyoccalyx are not available. Other methods rely on the release of glycocalyx constituents into the coronary circulation or on the post-mortem visualization of the endothelial glycocalyx by (electron)microscopy [44, 45].

## Conclusions

The compromised high fat diet-induced hyperemic coronary microvascular perfusion response, which is associated with early stages of insulin resistance, can be countered by treatment with both metformin and sulodexide. The adverse effect of acute glycocalyx degradation and protective effect of long-term sulodexide administration on myocardial perfusion provides indirect evidence pointing to a role of the glycocalyx in preserving myocardial microvascular function in pre-diabetic animals. Additional studies are warranted to fully delineate the role of the coronary microvasculature, and the endothelial glycocalyx in particular, in the development of cardio-metabolic disease.

## Abbreviations

Akt: protein kinase B
4CH: 4-chamber
dV/dt: change of left ventricular volume as a function of time
DS: dermatan sulfate
EDV: end-diastolic volume
EF: ejection fraction
EG: endothelial glycocalyx
ESV: end-systolic volume
FA: fatty acids
FFA’s: free fatty acids
GAG: glycosaminoglycan
HFD: high fat diet
IL-6: interleukin-6
HP: heparin
IPGTT: intraperitoneal glucose tolerance test
IVITT: intravenous insulin tolerance test
LA: long axis
LV: left ventricular
MRI: magnetic resonance imaging
NEFAs: non-esterified fatty acids
PBR: perfused boundary region
RBC: red blood cell
ROI: region of interest
SA: short axis
SDF: Sidestream Dark-Field
TE: echo time
TGF-β: transforming growth factor-β
TNF-α: tumor necrosis factor-alpha
TR: pulse repetition time
