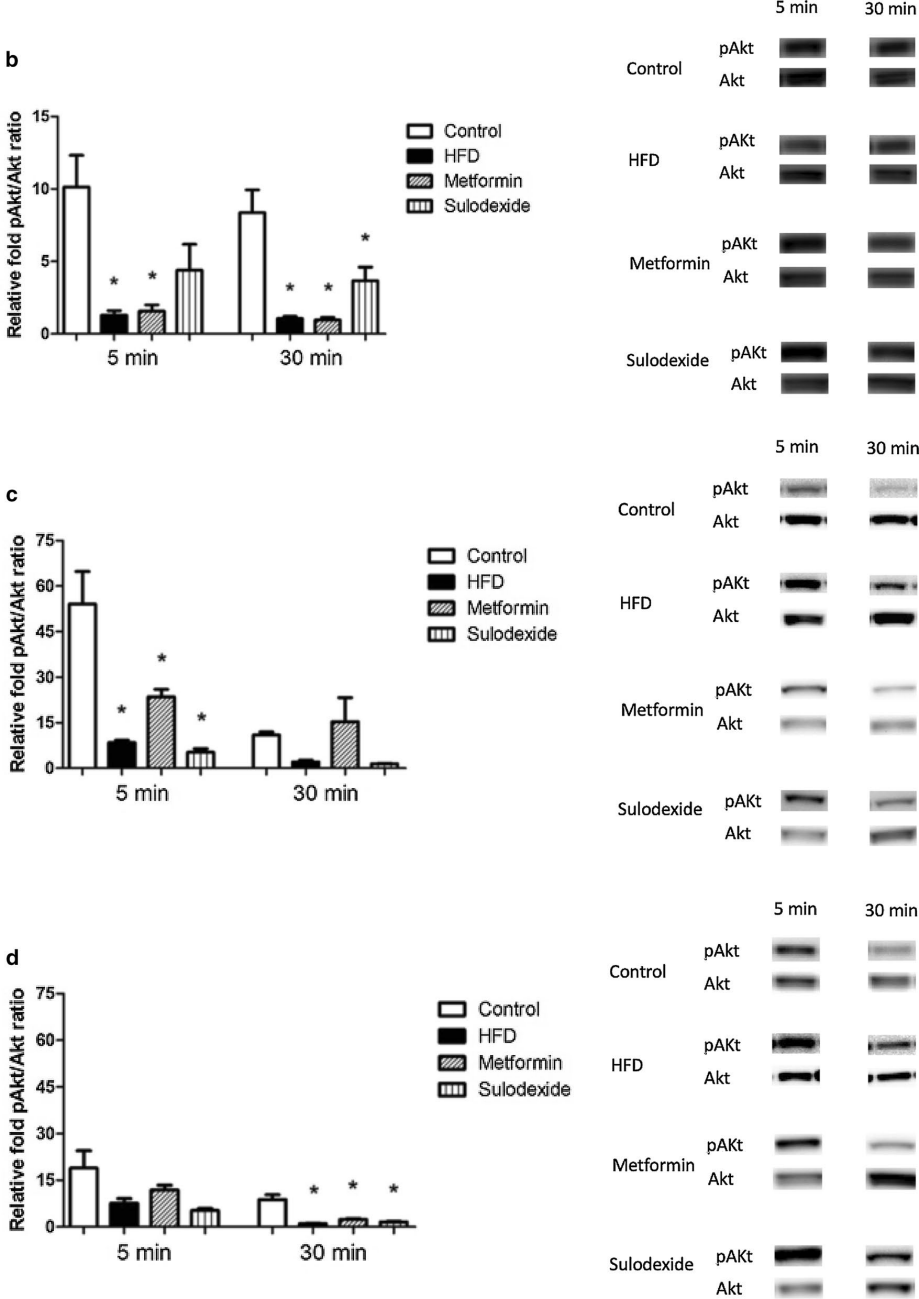# Correction to: Metformin and sulodexide restore cardiac microvascular perfusion capacity in diet‑induced obese rats

**DOI:** 10.1186/s12933-021-01240-7

**Published:** 2021-02-26

**Authors:** Judith van Haare, M. Eline Kooi, Jurgen W. G. E. van Teeffelen, Hans Vink, Jos Slenter, Hanneke Cobelens, Gustav J. Strijkers, Dennis Koehn, Mark J. Post, Marc van Bilsen

**Affiliations:** 1 Department of Physiology, Maastricht University, P.O. Box 616, 6200 MD Maastricht, The Netherlands; 2 Department of Radiology and Nuclear Medicine, Maastricht University, P.O. Box 616, 6200 MD Maastricht, The Netherlands; 3 Department of Cardiology, CARIM School for Cardiovascular Diseases, Maastricht University, P.O. Box 616, 6200 MD Maastricht, The Netherlands; 4 Biomedical Engineering and Physics, Academic Medical Center, P.O. Box 22700, 1100 DE Amsterdam, The Netherlands; 5 Pie Medical Imaging, P.O. Box 1132, 6201 BC Maastricht, The Netherlands

## Correction to: Cardiovasc Diabetol (2017) 16:47 https://doi.org/10.1186/s12933-017-0525-7

Following publication of the original article [1], the authors regret errors in Figs. 2b–d. In these figures the images of the representative Akt and phospho-Akt (pAkt) signals should be replaced with the appropriate images. The representative images shown here are correct. The changes do not affect the scientific conclusion and significance of the article.